# Datasets on the measurement of mechanical properties of ferrite and austenite constitutive phases using nanoindentation and micro hardness techniques

**DOI:** 10.1016/j.dib.2019.104551

**Published:** 2019-09-23

**Authors:** Ayorinde Tayo Olanipekun, Maledi Nthabiseng, Olusoji Oluremi Ayodele, M.R. Mphahlele, Bob Mpinda Mampuya, Peter Apata Olubambi

**Affiliations:** a Department of Mechanical Engineering Science, University of Johannesburg, South Africa; b Centre for Nano-Engineering and Tribocorrosion, School of Mining, Metallurgy and Chemical Engineering, University of Johannesburg, South Africa; c School of Chemical and Metallurgical Engineering, University of Witwatersrand, Johannesburg, South Africa

**Keywords:** Duplex stainless steel, Ferrite, Austenite, Nanoindentation, Microhardness

## Abstract

The major objective of this work is to study the hardness data at the domain of ferrite and Austenite phases. Nanoindentation and microhardness study has been conducted on austenite and ferrite present in the microstructure of hot rolled and heat treated duplex stainless steel (2205 DSS). Furthermore, Optical microscopy and field emission scanning electron microscope (FE-SEM) were used to identify the microstructural distribution and phases present. Austenite reveals higher nanohardness data value than ferrite, as oppose to ferrite average elastic modulus which is higher than that of austenite. Also, higher value of microhardness data was observed for austenite in comparison with the ferrite at different load application.

**Table:** Specifications Table

Subject	Mechanical Engineering and Materials science
Specific subject area	Metals and alloys, Nanotechnology
Type of data	Table Graph Figure
How data were acquired	- Phases were determined by image analysis taken by optical microscope (OM) (Model Axio observer 7 for materials, Carl Zeiss microscopy, GmbH, Germany). Also, phases present was obtained by field emission scanning electron microscope (FE-SEM) (model Carl Zeiss sigma, Germany) - X-ray diffraction of patterns of the as received hot rolled annealed sample was carried out by Xray diffractometer. The reflection peaks in as received sample shows the presence of two phases only: δ−ferrite and γ−Austenite phases with (110) and (111) as the major reflection peaks, respectively. The peak analysis was carried out using (PDXL software). - The experimental datasets were obtained through the experiment that was carried out on an ultra-nanoindenter (UNHT), manufactured in Switzerland, equipped with a three-sided pyramid, berkovich diamond indenter. However, all the indentation tests followed ISO 14577 - The Vickers microhardness (HV) was measured by Vickers microhardness tester (FUTURE-TECH FM 800) at a load (P) 20 gf (1.0 N)- 200gf and dwell time of 10 s at room temperature, with five repeat tests to ensure data reliability
Data format	Raw Analyzed and Filtered
Parameters for data collection	- For the indentation test, the total indentation time is 40s which was divided using load control function with a 20s loading time, 5s holding time and 15s unloading time. However the load was vary from 30mN to 50mN for the test. - For the Vickers hardness test, load was varied from 20g, 50g,100g,200g on both austenite and ferrite phases.
Description of data collection	A sequence of microhardness test under load ranging from 20g to 200g, while the load span for the nanoindentation test was from 30 mN to 50mN. Local hardness mechanical property of the austenitc and ferritic phase in a commercial hot rolled and annealed DSS (2205) was measured. The micro hardness data presented for the austenite phase at each successive load is higher than of the ferritic phase. Likewise, the average nanohardness was higher for austenitic phase (8.9GPa) when compared to ferrite phase (7.9GPa). Annealing heat treatment has been suggested as the reason why there is diversity in the hardness value of the ferritic phase and the austenitic phase
Data source location	University of Johannesburg and University of Witwatersrand Johannesburg South Africa
Data accessibility	With the article

**Table:** 

**Value of the Data** • The data gotten can be used in the Industries to determine the mechanical properties of metallic alloy. • The methodology, data, and the techniques used in analyzing, can be easily replicated by other researchers at different laboratories for further insights and development of experiments. • A mechanical characterization technique has been presented, which can be used to determine nanohardness and vickers hardness of Duplex stainless steel experimentally. • Future research on nanoindentation analysis can be built on the work done.

## 1 Data

**Fig. 1:**
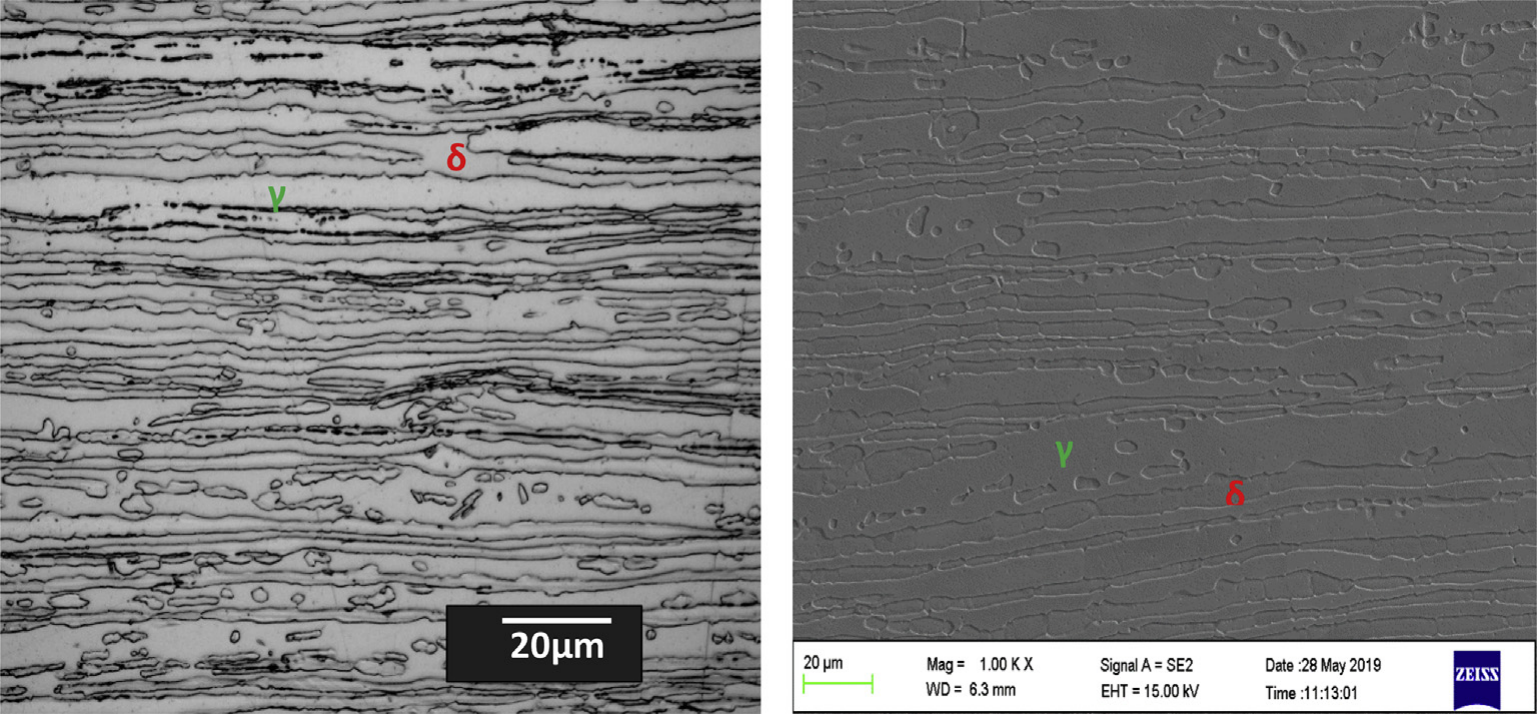
(a) OM micrograph (b) SEM micrograph of the as received hot rolled DSS, showing austenite and ferrite phase.

**Fig. 2:**
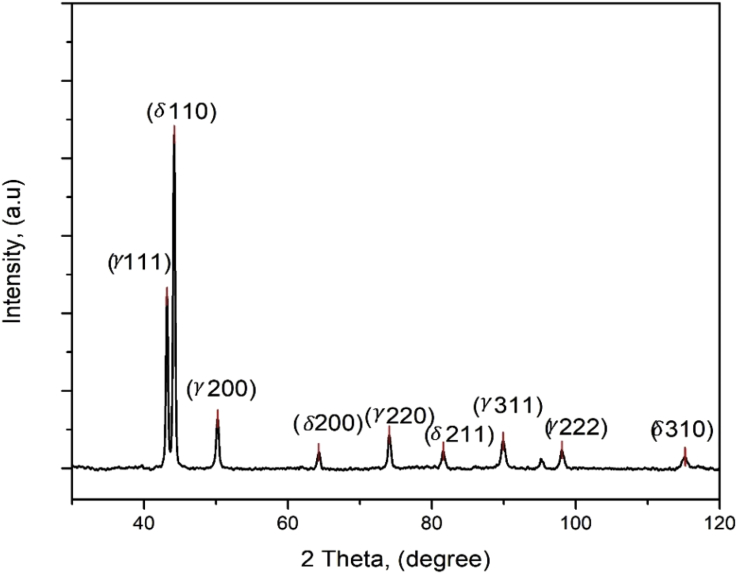
An X-Ray diffraction (XRD) pattern analysis of the as received annealed and hot rolled DSS 2205.

**Fig. 3:**
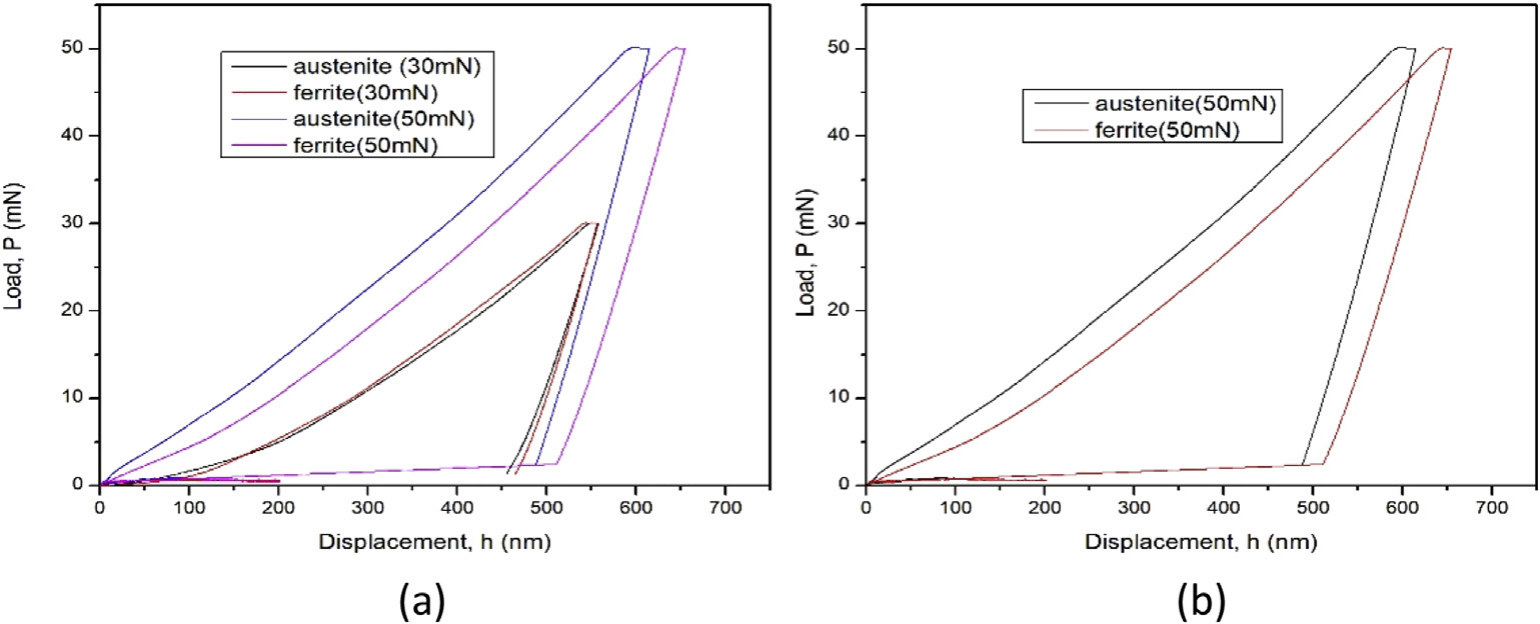
Nanoindentation Load-displacement curves (a) at load 30Mn and 50Mn (b) at 50mN.

**Fig. 4:**
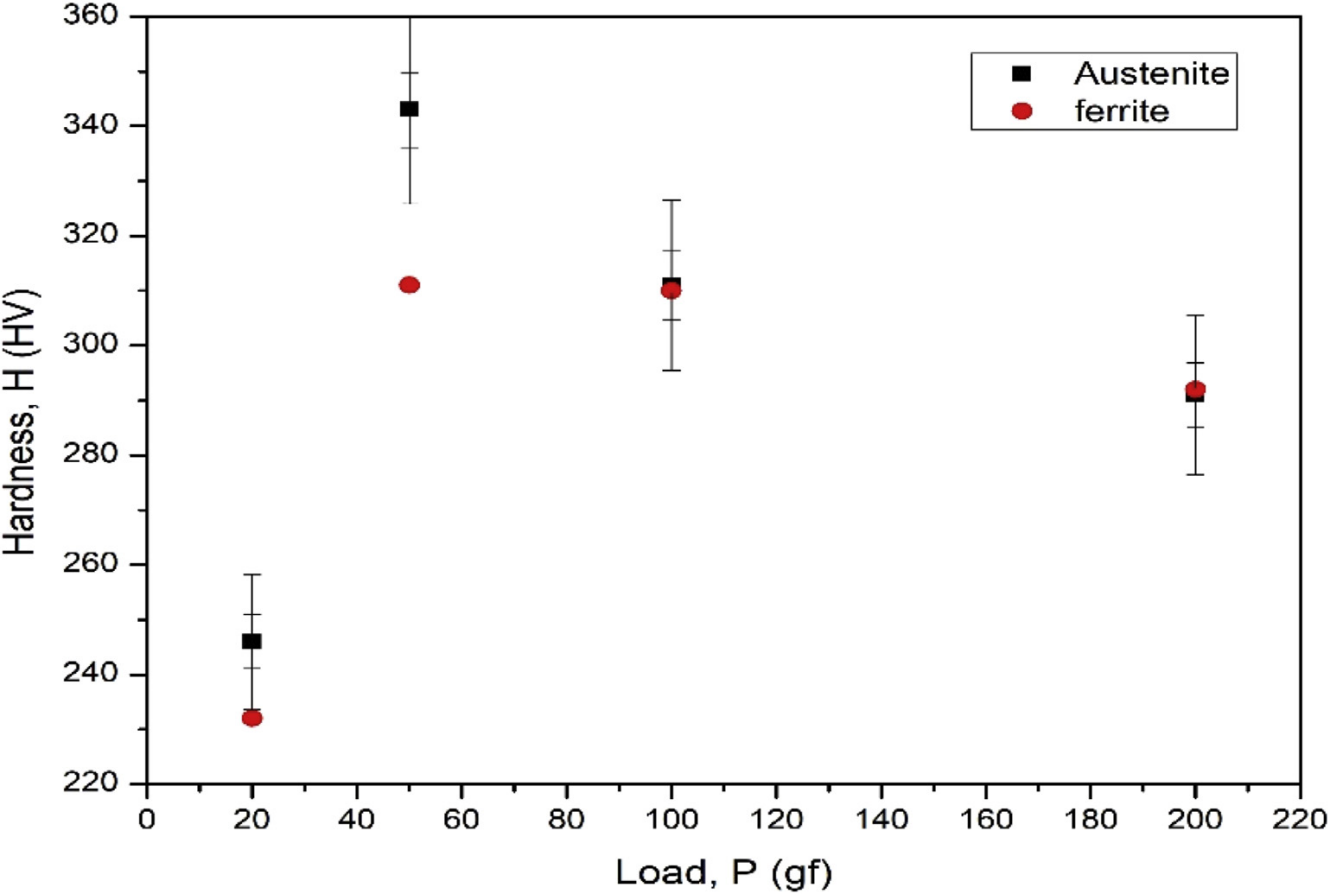
Vickers microhardness values for ferrite and Austenite.

The dataset in this article describes the microstructure and mechanical analysis of DSS (2205). The chemical composition data analysis of the as received DSS (2205) is shown in Table 1. Fig. 1 (a) and (b) describe the OM and SEM characterization of the as-received hot rolled heat treated 2205 DSS respectively, revealing the austenite and ferrite phases. Fig. 2 describes the XRD characterization of the 2205 DSS. Fig. 3 Describes the nanoidentation load displacement curves for ferrite and austenite captured at various loads, varied between 30mN and 50mN. The mean value data of the nanohardness, reduced modulus and modulus calculated from the nanoidentation experiment (Table 2). The Vickers hardness for the respective phases (Table 3). Fig. 4 describes Vickers microhardness values conducted at the austenite and ferrite interface.

**Table 1:** Chemical composition of the as received hot rolled, annealed duplex stainless steel (2205) (wt %).

C	S	P	Mn	Si	Cr	Ni	Mo	N
0.023	0.001	0.022	1.38	0.56	22.8	5.3	3.37	0.16

**Table 2:** Hardness and modulus from nano-indentation experiments.

Phase	Hardness, $\bar{H}\left(GPa\right)$	Reduced modulus $\bar{E_{r}}\left(GPa\right)$	Modulus, $\bar{E}\left(GPa\right)$
Ferrite	7.9	163.8 0	178.84
Austenite	8.9	137.28	149.89

**Table 3:** Vickers Hardness for the respective phases.

Load (gf)	Austenite Hardness (HV)	Ferrite Hardness (HV)
20	246	232
50	343	311
100	311	310
200	291	292

## 2 Experimental design, materials, and methods

### 2.1 Materials and metallographic preparation

The material investigated in this research was obtained from Columbus stainless (pty) Ltd. as 6mm × 1500mm × 6000mm rectangular sheet, hot rolled and annealed 2205 DSS at 1050 °C–1100 °C then quenched in air and water spray. The samples were prepared following the standard metallographic technique, first polished followed by electro-chemical etching using KOH etchant solution. Phases were determined by image analysis taken by optical microscope (OM) (Model Axio observer 7 for materials, Carl Zeiss microscopy, GmbH, Germany). Also, the phases present was obtained by field emission scanning electron microscope (FE-SEM) (model Carl Zeiss sigma, Germany).

### 2.2 Nanoindentation

The nanoindentation was carried out on an ultra-nanoindenter (UNHT), manufactured in Switzerland, equipped with a three-sided pyramid, berkovich diamond indenter. All the indentation tests followed ISO 14577. Before the indentation tests, the contact area was calibrated by an indirect method to maintain accuracy. The total indentation time is 40s which was divided using load control function with a 20s loading time, 5s holding time and 15s unloading time. Also, varying load from 30mN to 50mN, and the grid of indents was spaced 2 μm apart with four different indentation points in each phase.

According to Tao et al. [1] contact area can be expressed as the equation below,

(1) $$A_{c}=C_{0}h_{c}^{2}+C_{1}h_{c}+C_{2}h_{c}^{\frac{1}{2}}+C_{3}h_{c}^{\frac{1}{4}}+C_{2}h_{c}^{\frac{1}{2}}+\ldots \;$$

From Kicks Law [2],

(2) $$P=Ch^{2}$$

Showing that the load is directly proportional to the square value of indentation displacement. WherePis the indentation load and C is the loading curvature, while h is the displacement. Elastic modulus and nanohardness was determined by the following equations from (3), (4), (5) proposed by Oliver-Pharr [3], [4].

(3) $$H=\frac{P_{max}}{A_{c}}\;$$

(4) $$E_{r}=\frac{\sqrt{\pi }S}{2\sqrt{A_{c}}}\;$$

(5) $$\frac{1}{E_{r}}=\frac{1-\vartheta^{2}}{E}+\frac{1-\vartheta_{i}^{2}}{E_{i}}$$

(6) $$S=\frac{dP}{dh}$$

The initial unloading curve$\;\frac{dP}{dh}$ is defined by S which is the stiffness, while H is nanohardness, maximum force is denoted by $P_{max}$, while the contact area is denoted by $A_{c}$. $E_{r\;}$is the reduced modulus, the elastic modulus and the Poisson's ratio respectively for the diamond indenter are $E_{i}=1140Gpa$ and. $\vartheta_{i}=0.07$

Fig. 1 is a schematic representation of nanoindentation head, while Fig. 2, is a scheme of indentation curve clearly showing the energies $E_{t},E_{p},E_{e}$ and the maximum (hmax) depths and residual (hf).

The indentation hardness and elastic modulus was obtained from Oliver -Pharr analysis [4] as shown in equations (3), (4), (5), (6). However, we observed that the ferrite elastic modulus (178.84GPa) is higher than austenite elastic modulus(149.89GPa), close to the data obtained by Karim et al. [5]. Similarly, Moverare and Oden [6] explained in their work that ferrite always have the strongest phase, Inal et al. [7] defer in his own explanation, saying DSS tend to behave contrary, presenting stronger austenite phase than the ferrite phase. This fact was substantiated by Meshkov and Pereloma [8] that fine grained austenite with uniform grain size after quenching and rapid annealing allows the realization of high strength in steel. In the same vein, the residual stresses among the DSS phases generated by the heat treatment can also be responsible for the disparity in the hardness value of the constituent phases. Likewise, Moverare and Oden observed that “Nitrogen acts a austenite phase stabilizer and also promote planar glide which strengthens austenite” [6] and the percent of Ni in our alloy is 5.3 which is high enough to stabilize the austenitic phase after heat treatment.

### 2.3 Microhardness test

The Vickers microhardness (HV) was measured by Vickers microhardness tester (FUTURE-TECH FM 800) at a load (P) 20 gf (1.0 N)- 200gf and a dwell time of 10 s at room temperature, with five repeat tests to ensure data reliability.

The Vickers microhardness test was conducted on both the ferrite and austenite phases. Meanwhile, the Vickers microhardness experimental data, indicated that there was no significant difference between the hardness value of the austenite phase and the ferrite phase except for the hardness test carried out at 50g load giving an average hardness values of 343 HV and 311 HV for ferrite. However, we can conclude and assume that, the factors responsible for high Nano hardness can also be the same factors for austenite high micro hardness.
